# Impact of asthma on mouth breathing, occlusion and salivary parameters in a group of school-aged children: a cross-sectional study

**DOI:** 10.1186/s12903-026-08537-7

**Published:** 2026-05-18

**Authors:** Zainab H. J. ALQassab, Engy Saad Elkaragy, Reham S. Soliman, Karin M. L. Dowidar

**Affiliations:** 1 Department of Pediatric Dentistry and Dental Public Health, Faculty of Dentistry, Alexandria University, Alexandria, Egypt; 2 Department of Pediatrics, Faculty of Medicine, Alexandria University, Alexandria, Egypt

**Keywords:** Asthmatic children, Mouth breathing, Dental occlusion, Salivary parameters

## Abstract

**Background:**

Children with asthma may experience functional and developmental oral changes due to altered breathing patterns and systemic effects of the disease. This study aimed to evaluate the relationships among mouth breathing, occlusion, and salivary parameters in school-aged children with asthma.

**Methods:**

This comparative cross-sectional study included 88 children who were equally divided into two groups. Group I (study group): This group consisted of children with asthma who were sex- and age-matched. Group II (the control group): included healthy children. The ages ranged from 6–12 years. An interview-based questionnaire, clinical examination, and salivary biochemical studies were conducted.

**Results:**

This study revealed a significantly greater prevalence of mouth breathing in asthmatic children (*p* < 0.001), especially in patients with uncontrolled asthma. Additionally, they had a greater prevalence of anterior overjet (*p* = 0.01) and anterior open bite (*p* = 0.02). Interestingly, none of the children presented a posterior crossbite. Notably, no significant difference in salivary parameters was observed between the two groups.

**Conclusion:**

Asthma and its level of control may influence functional and developmental aspects of oral health, particularly in children with mouth breathing. Early and effective asthma management, along with monitoring oral functions, can help mitigate these effects and support better overall oral health in asthmatic children.

**Supplementary Information:**

The online version contains supplementary material available at 10.1186/s12903-026-08537-7.

## Background

Asthma is a chronic respiratory condition characterized by inflammation and narrowing of the airway, leading to symptoms such as wheezing, coughing, chest tightness, and dyspnea [1].Globally severe wheezing episodes affect about 20% of children aged 6–7 years each year [2], while in Egypt, asthma impacts more than 7.7% of the pediatric population [3].

The primary goals of asthma therapy focus on improving the quality of life of children, mainly by allowing them to engage in normal activities, maintain normal pulmonary function, and avoid adverse effects from medications [4, 5]. Asthma symptoms are mainly controlled by bronchodilators and anti-inflammatory agents [3, 5, 6], with treatment initiated on the basis of the frequency and severity of asthma symptoms [3, 4].

One of the most common oral manifestations of asthma is mouth breathing, which is often adopted as a compensatory mechanism due to airflow obstruction or nasal congestion, with a prevalence ranging from 31% to 42.8% in asthmatic children, which is markedly higher than that in the general pediatric population [7–9]. This altered breathing pattern has been associated with anterior gingivitis, increased plaque accumulation, and malocclusion [10–13].

Moreover, altered breathing patterns in asthmatic children have been associated with malocclusion, as they disturb the balance of the orofacial musculature, impair maxillary bone growth, and affect dentoalveolar development, ultimately leading to skeletal discrepancies [14, 15]. This highlights the importance of recognizing asthma not only as a respiratory disorder but also as a condition with potential long-term implications for craniofacial growth.

In addition to its skeletal and occlusal effects, long-term mouth breathing is associated with salivary function [14]. Reduced nasal breathing can impair salivary gland stimulation, leading to changes in flow rate, pH, and buffering capacity [16, 17]. Saliva is central to maintaining oral homeostasis through antimicrobial activity, lubrication, remineralization, and neutralization of acids [18]. Alterations in these parameters compromise the protective role of saliva.

Asthma itself, as well as its pharmacological management (notably β₂-agonists and inhaled corticosteroids), may further influence salivary characteristics. These medications have been associated with reduced salivary flow, lower pH, and altered buffering capacity [19–23]. These factors may affect the antimicrobial activity, lubrication, and overall oral homeostasis of saliva [18]. Additionally, oxidative stress (OS) in asthma may disrupt the salivary antioxidant system, with total antioxidant capacity (TAC) serving as a biomarker of this imbalance [23–28].

Unlike the majority of previous studies that have investigated the associations between asthma and common oral diseases such as dental caries, erosion, or periodontal conditions [29–31], the present study did not assess these outcomes. Instead, it focuses on other underexplored oral consequences of asthma, namely, mouth breathing, occlusal development, and salivary characteristics. These variables were selected because they reflect functional and developmental aspects of oral health rather than direct risk indicators for caries. Understanding these functional and developmental oral changes is critical for optimizing clinical management and promoting long-term oral health in asthmatic children.

Therefore, this comparative cross-sectional study aimed to evaluate mouth breathing, occlusion, and salivary parameters in asthmatic school-aged children compared with healthy controls. The null hypothesis was that asthma does not significantly influence the prevalence of mouth breathing, occlusal characteristics, or salivary parameters in school-aged children.

## Materials and methods

### Ethical approval and informed consent

Ethical approval for the study was obtained from the Research Ethics Committee of Alexandria University, Faculty of Dentistry (IRB 00010556–IORG 0008839). The research followed the Declaration of Helsinki [32] and was reported according to the STROBE guidelines [33]. Written informed consent to participate in the study was obtained from the parents or legal guardians of all participants prior to examination and saliva sample collection.

### Study design

This observational, comparative cross-sectional study was conducted at the clinic of the Department of Pediatric Dentistry and Dental Public Health, Alexandria University, Egypt, from 2023—2024. Children with asthma were recruited from Alexandria University Children’s Hospital (AUCH) and the Pediatric Dental Clinic in the Department of Pediatric Dentistry and Dental Public Health, Faculty of Dentistry, Alexandria University.

### Sample size calculation

**Fig. 1:**
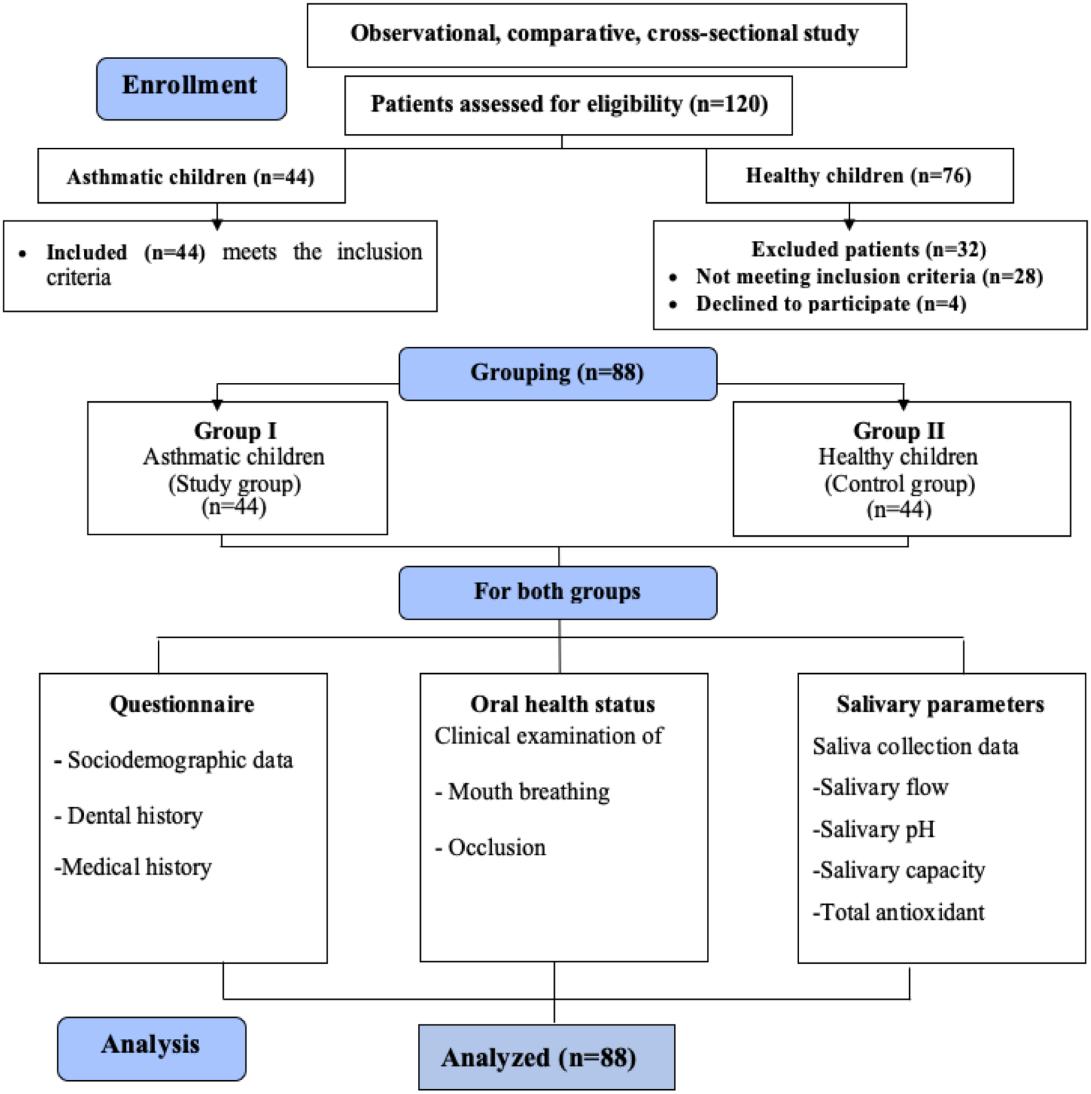
Flow chart of the participants and groups

The sample size was calculated based on the primary outcome of mouth breathing, using proportions reported by dos Santos et al. [9] (66.3% in asthmatic children vs 33.8% in non-asthmatic children) corresponding to an effect size of 32.5%. Assuming a 5% alpha error and 80% study power. On the basis of a comparison of proportions, the minimum sample size was calculated to be 36 children per group, which was increased to 44 to compensate for nonresponse. The total required sample size = Number of groups × number per grou*P* = 2 × 44 = 88 children. The sample size was calculated via MedCalc Statistical Software version 19.0.5 (MedCalc Software bvba, Ostend, Belgium; https://www.medcalc.org; 2019) [34]. (Fig. 1).

### Patient selection

A total of 88 children aged 6–12 years whose parents or legal guardians provided written informed consent were included in the study. The case group consisted of 44 children who had been previously diagnosed with asthma by a pediatric respiratory specialist and were under regular treatment with medications such as inhaled corticosteroids, long-acting beta-agonists (LABAs), short-acting beta-agonists (SABAs), montelukast, or systemic steroids [35]. The duration of asthma varied among children, but all had been on regular medications for at least one year. For each child, the type, dosage, and duration of medications and inhalation technique were recorded, noting that dosages could change based on clinical needs. These children were recruited from Alexandria University Children's Hospital (AUCH). The control group included 44 healthy children who were free of any medical conditions and were selected from asthmatic children’s siblings, if available, or from children attending the Pediatric Dental Clinic, as well as siblings from the Pediatric Dentistry and Dental Public Health Department, Faculty of Dentistry, Alexandria University.

Children with serious illnesses or chronic systemic conditions, such as diabetes mellitus, cardiac diseases, renal disorders, and other conditions that could affect mouth breathing, including adenoid hypertrophy, chronic nasal obstruction, or allergic rhinitis, were excluded from the study. Additionally, children undergoing orthodontic treatment were excluded [3, 35, 36].

### Training, calibration, and examiner reliability

Mouth breathing was assessed using a mirror test by a single trained examiner. Twenty percent of participants were re-evaluated after 2 weeks, and intra-examiner reliability was (Cohen’s κ = 0.85), Near-perfect agreement [37]. Inter-examiner reliability was not applicable.

### Data collection methods

#### Questionnaire

Data were obtained through an interview-based questionnaire administered to parents or caregivers. The questionnaire was developed by the authors for the purpose of this research. Some items were adapted from previously published studies [36, 38–40], and the medical history section was developed according to international asthma management guidelines. The full English version of the questionnaire is provided as Supplementary file 1.

The questionnaire assessed several domains, including the child’s sociodemographic characteristics (age, sex, parental education, and occupation) and history of dental visits (dental visits in the last year, reasons for the dental visit) [36, 38–40]. Medical history related to asthma, including the level of asthma control, was documented using a validated four-item symptom questionnaire according to the Global Initiative for Asthma (GINA) guidelines [5], which assessed daytime symptoms, night waking, use of short-acting beta-agonists (SABA), and activity limitation. Responses were combined as follows: none of the four items positive = well controlled; 1–2 items positive = partially controlled; 3–4 items positive = uncontrolled. Information on the current medication regimen (type, dosage, and duration) and inhalation technique was also recorded. Additionally, data on associated morbidities and exposures were collected, including exposure to smoking, symptoms of allergic rhinitis, eczema, food allergies, use of proton pump inhibitors (PPIs), and patterns of mouth breathing or snoring during sleep (whether transient during colds or persistent). Further environmental exposures, such as contact with industrial materials, noxious chemicals, dust, and domestic animals or pets, were also noted [5].

#### Intraoral examination for mouth breathing and occlusion

A mirror test was used to detect mouth breathing. A double-sided mirror was held between the nose and mouth. If there is fogging on the mouth side of the mirror, the patient is considered a mouth breather, and if the fogging towards the nasal side, it indicates nasal breathing [41]. In addition to the mirror test, parental reports of nocturnal mouth breathing patterns were collected via the validated questionnaire to capture habitual mouth breathing.

Occlusion was clinically assessed with participants seated in an upright position with the head in a natural posture. Anterior and posterior relationships were evaluated using a sterile periodontal probe and dental mirror. Anterior assessments included assessments of overjet, overbite, open bite, and canine relationships (Cl I (Normal), Cl II (Overbite/Distocclusion), and Cl III (Underbite/Mesiocclusion)). Posterior assessment involved classification of molar relationships according to the terminal plane in primary dentition (Flush, Mesial, Distal relation), and Angle’s classification in mixed or permanent dentition (Cl I as the upper mesiobuccal cusp fitting the lower buccal groove, while Cl II (mandible retruded) and Cl III (mandible protruded)). Posterior crossbite was also recorded when present. Registrations were considered not applicable when relevant teeth were missing or not fully erupted [42].

#### Salivary collection and biochemical analysis

Unstimulated saliva, reflecting resting glandular function, was collected at least two hours after breakfast or tooth brushing, with participants spitting into sterile tubes. The salivary flow rate was assessed (ml/2 min). Saliva was collected for exactly 2 min in a graduated tube to calculate the initial flow rate (ml/min). A total of 5 ml of saliva was collected for biochemical analysis [36]. The collected saliva was immediately transferred into a sterile, labelled container and stored in an ice box maintained at 4 °C with gel packs. The samples were transported to the Biochemistry Laboratory within two hours to preserve their integrity for pH, buffering capacity, and total antioxidant capacity (TAC) analysis.

In the biochemistry laboratory, a digital portable pH meter (Hanna Instruments® Microprocessor pH Meter pH 211, Woonsocket, RI, USA) was used to measure the salivary pH, the electrode was submerged in a test tube containing 2 ml of the saliva sample, and the reading was recorded once it stabilized [36]. Titration of 0.1 ml of 0.01 N HCl solution was used to determine the buffering capacity [36]. Saliva was frozen at −80 degrees Celsius until TAC analysis, which was conducted via an antioxidant colorimetric assay kit (Biodiagnostic, Dokki, Giza, Egypt) and a spectrophotometer to evaluate the optical absorption of the sample [27].

### Outcome assessment

The outcomes of this study included the presence of mouth breathing [41], occlusal characteristics (overjet, overbite, open bite, posterior crossbite, and molar and canine relationships) [42], and salivary parameters (flow rate, pH, buffering capacity) [27–36]. Independent variables included medical history (e.g., asthma status) and dental history [5, 37]. Potential confounders, such as age, sex, and parental education level, were also considered in the analysis because of their possible influence on oral and salivary outcomes [36, 39, 40].

### Statistical analysis

Data were analyzed using IBM SPSS Statistics version 23.0, with significance set at *p* < 0.05. Continuous variables were summarized as mean (SD) or median (IQR), and categorical variables as frequencies and percentages. Group comparisons were performed using t-tests or Mann–Whitney U tests for continuous variables and chi-square tests for categorical variables. Pearson correlation was used to assess associations between asthma medications and salivary parameters.

A multivariable binary logistic regression using the Enter (forced entry) method was performed to evaluate factors associated with mouth breathing. The dependent variable was coded as 1 = presence and 0 = absence of mouth breathing, so adjusted odds ratios represent the odds of mouth breathing. Covariates were selected a priori based on clinical relevance. Independent variables included sociodemographic, child gender, asthma history, medication regimen, and asthma control.

Multicollinearity was checked (VIF < 5). Model fit was assessed with the Hosmer–Lemeshow test (χ^2^ = 10.60, *P* = 0.23), − 2 log likelihood = 42.79, and Nagelkerke’s R^2^ = 0.37. Potential confounders (e.g., parental education) were considered, but limited events precluded their inclusion to avoid overfitting; this is discussed in the Discussion section. Adjusted odds ratios (AORs) with 95% CIs were reported.

## Results

Total of 88 children aged 6–12 years were enrolled and distributed into two groups: asthmatic patients (n = 44) and healthy controls (n = 44). A table including participants’ sex, age, and a unique code for each child (asthmatic children coded A01–A44, and healthy controls coded H01–H44) was used to facilitate matching. All asthmatic children were selected first randomly, followed by their healthy siblings when available, and each was then matched to a healthy control based on age (in years) and sex to ensure comparability (Fig. 1). The median age in both groups was 8 years, with no significant differences in age or sex distribution (*P* = 1.00). However, parental education levels differed significantly, with higher education being more prevalent among parents of asthmatic children (*P* = 0.01).

Dental attendance within the past year was significantly greater among healthy children than among asthmatic children (52.3% vs. 22.7%, *P* < 0.01). In both groups, the most common reasons for dental visits were pain relief and treatment needs.

**Fig. 2:**
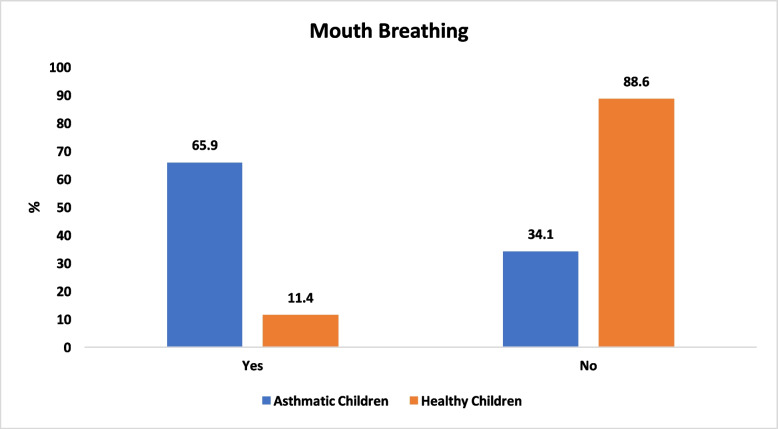
Comparison of mouth breathing between the study groups

Mouth breathing was significantly more common in asthmatic children (65.9%) than in healthy controls (11.4%) (*P* < 0.001; Fig. 2). The occlusal evaluation revealed that asthmatic children had greater overjet (median: 3 mm vs. 2 mm, *P* = 0.01), whereas anterior open bite was more common among asthmatic children (13.6% vs. 0%, *P* = 0.02; Table 1). Additionally, Angle’s Class II malocclusion was more prevalent in asthmatic children (25% vs. 6.8%, *P* = 0.02; Table 1). No significant differences in overbite (*P* = 0.39), posterior crossbite (*P* = 1.00), canine relationships (*P* = 0.71, *P* = 0.35), terminal plane relationships (*P* = 0.36, *P* = 0.35), or molar classifications (*P* = 0.86, *P* = 0.69; Table 1) were detected between groups.

**Table 1:** Comparison of mouth breathing and occlusion between the study groups

			**Asthmatic Children** **(*n* = 44)**	**Healthy Children** **(*n* = 44)**	***P* value**
Mouth Breathing	Yes: n (%)		29(65.9)	5(11.4)	< 0.001*
	No: n (%)		15(34.1)	39(88.6)	
Occlusion (Anterior Relations)	Overjet Median (IQR)		3(3)	2(2.8)	0.01*
	Overbite Median (IQR)		30(40)	30(45)	0.39
	Anterior open bite	Yes: n (%)	6(13.6)	0(0)	0.02*
		No: n (%)	38(86.4)	44(100)	
Occlusion (Right cuspid Relation)	CL I: n (%)		32(72.7)	38(86.4)	0.02*
	CL II: n (%)		11(25)	3(6.8)	
	CL III: n (%)		1(2.3)	0(0)	
Occlusion (Left Cuspid Relation)	CL I: n (%)		36(81.9)	34(77.3)	0.71
	CL II: n (%)		5(11.4)	4(9.1)	
	CL III: n (%)		2(4.5)	3(6.8)	
Left Terminal plan	Mesial step: n (%)		9(20.5)	9(20.5)	0.36
	Flushed: n (%)		0(0)	2(6.8)	
Right Terminal plan	Mesial step: n (%)		8(18.2)	7(15.9)	0.35
	Flushed: n (%)		0(0)	2(6.8)	
Right Angle's classification	CL I: n (%)		28(63.6)	26(59.1)	0.86
	CL III: n (%)		8(18.2)	8(18.2)	
Left Angle's classification	CL I: n (%)		9(20.5)	23(52.3)	0.69
	CL III: n (%)		7(15.9)	27(61.4)	
Posterior cross bite	Yes: n (%)		0	0	––
	No: n (%)		44(100)	44(100)	

*IQR* Interquartile Range

^*^Statistically significant at *P* < 0.05

**Fig. 3:**
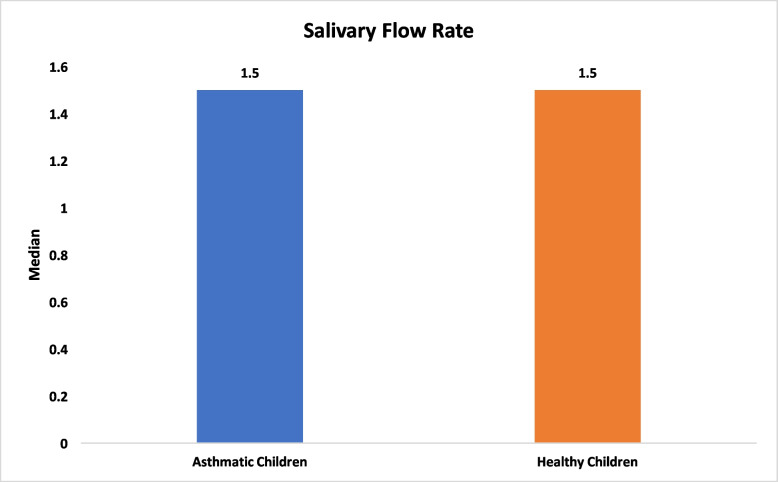
Comparison of salivary flow between healthy and asthmatic children

**Fig. 4:**
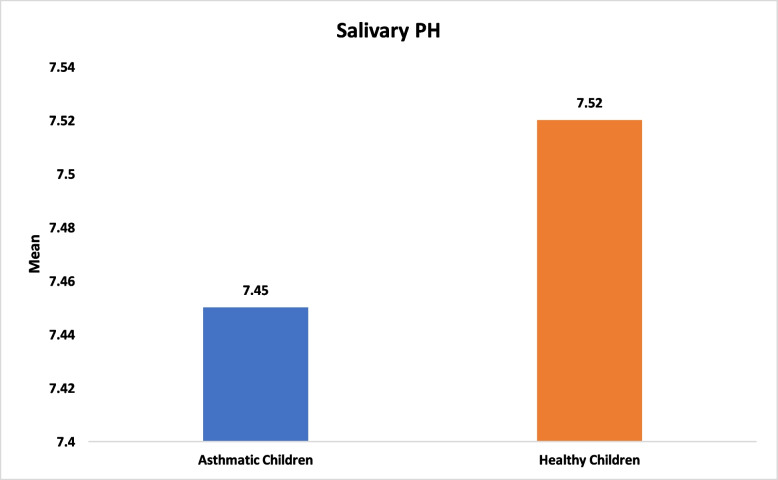
Comparison of salivary pH between healthy and asthmatic children

**Fig. 5:**
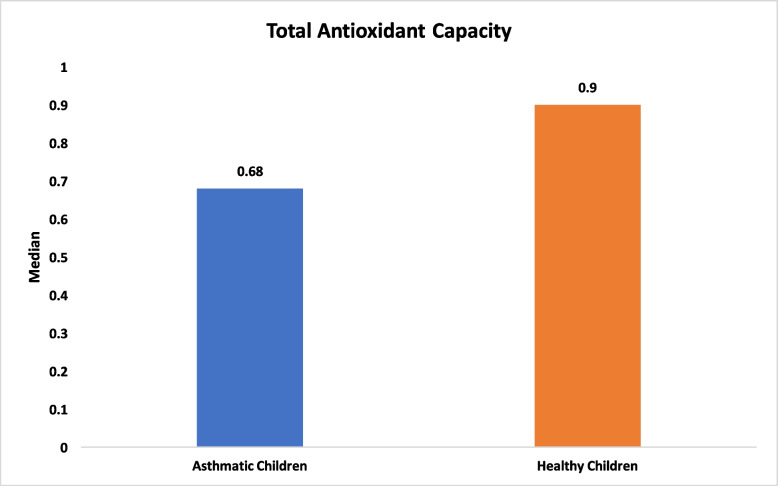
Comparison of total antioxidant capacity between healthy and asthmatic children

**Fig. 6:**
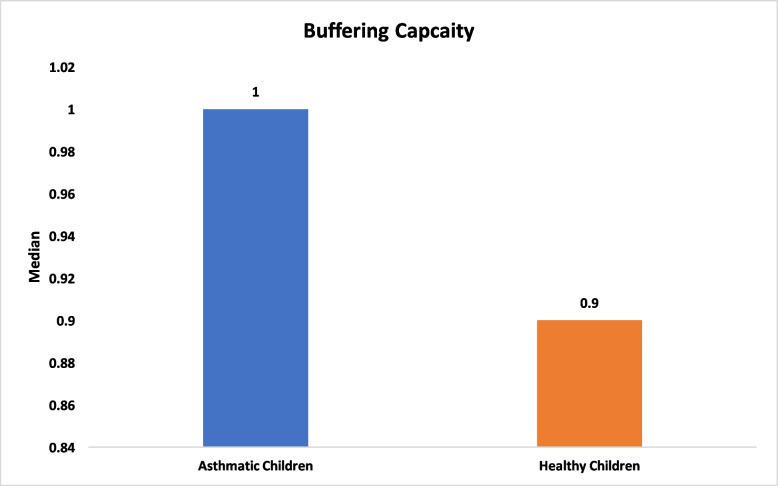
Comparison of buffering capacity between healthy and asthmatic children

With respect to salivary characteristics, no significant differences were found between asthmatic and healthy children in salivary flow rate (median 1.50 ml/2 min [IQR 0.75] vs. 1.50 ml/2 min [IQR 0.40], *P* = 0.48), pH (mean 7.45 vs. 7.52, *P* = 0.44; Figs. 3 and  4). Total antioxidant capacity (TAC) was similar between groups, with a median of 0.68 mM/L (IQR 0.76) in asthmatic children and 0.90 mM/L (IQR 0.98) in healthy children (*P* = 0.11; Fig. 5). Salivary buffering capacity was assessed by titration, quantified as the total volume of 0.01 N HCl required to reduce 1 ml of saliva to pH 5.0; the median buffering capacity was 1 ml (IQR 0.68) in asthmatic children and 0.90 ml (IQR 0.60) in healthy children, with no statistically significant difference between groups (*P* = 0.12; Fig. 6). No significant correlations were found between salivary parameters and the use of inhaled corticosteroids or montelukast (*P* > 0.05; Tables 2 and 3).

**Table 2:** Correlations between inhaled corticosteroid use and salivary parameters in asthmatic children

	Pearson’s Correlation	***P* value**
Salivary flow ml/2 min	−0.11	0.47
Salivary PH	0.17	0.23
Volume of NHcl PH 7.00 in μl	0.14	0.38
Volume of NHcl PH 6.00 in μl	0.03	0.87
Volume of NHcl PH 5.00 in μl	0.12	0.42
Total Volume of NHcl in ml	0.14	0.38
Total antioxidant capacity (TAC) mM/L	−0.11	0.47

*TAC* Total antioxidant capacity

^*^Statistically significant at *P* < 0.05

**Table 3:** Correlations between montelukast use and salivary parameters in asthmatic children

	Pearson’s Correlation	***P* value**
Salivary flow ml/2 min	−0.06	0.72
Salivary PH	0.21	0.18
Volume of NHcl PH 7.00 in μl	0.23	0.13
Volume of NHcl PH 6.00 in μl	0.05	0.73
Volume of NHcl PH 5.00 in μl	0.02	0.89
Total Volume of NHcl in ml	0.16	0.30
Total antioxidant capacity (TAC) mM/L	−0.21	0.16

TAC: Total antioxidant capacity

^*^Statistically significant at *P* < 0.05

Uncontrolled asthma was significantly associated with an increased likelihood of mouth breathing (AOR = 20.0, 95% CI: 1.39–333.3, *P* = 0.03; Table 4). No other sociodemographic or clinical factors showed a statistically significant association.

**Table 4:** Regression model for factors affecting mouth breathing in asthmatic children

Explanatory variables		AOR (95% CI)	*P* value
**Sociodemographic background**			
Child gender	Male^a^	1	0.31
	Female	0.42 (0.08–2.33)	
**Asthma History**			
Medications	ICS + SABA + Montelukast	0.48 (0.04–5.00)	0.53
	ICS + SABA	1.45 (0.15–14.30)	0.74
	Montelukast alone or + ICS Or SABA^a^	1	
Asthma control status	Uncontrolled	20.00 (1.39–333.33)	0.03*
	Partially controlled	1.47 (0.17–12.50)	0.72
	Well controlled^a^	1	

*AOR* adjusted odds ratio, *CI* confidence interval, a: reference category, *ICS* Inhaled corticosteroids, *SABA* Short-acting beta agonist

−2 Log Likelihood: 42.79, Hosmer and Lemeshow Test X^2^: 10.60, *p* = 0.23, Nagelkerke’s R^2^ = 0.37

## Discussion

The present study sought to investigate the broader impact of asthma on oral health, extending beyond caries and hygiene to include mouth breathing, occlusal development, and salivary characteristics. Notably, there was a significant association between asthma and mouth breathing, which in turn was related to occlusal disturbances.

The asthma level of the asthmatic children was classified according to the Global Initiative for Asthma (GINA) 2023 guidelines [5]. A validated four-item symptom questionnaire was used [5]. Based on the responses, most asthmatic children (43.18%) were classified as having uncontrolled asthma, while 25.00% were well-controlled, and the remainder were partially controlled.

Mouth breathing was significantly more prevalent in asthmatic children. However, it should be noted that the assessment of oral breathing using the mirror test and questionnaire may be subject to misclassification bias due to the lack of objective diagnostic methods. While no significant association was found between mouth breathing and the medication regimen, a strong association was observed between mouth breathing and asthma control status, with a higher prevalence in children with uncontrolled asthma. This finding is consistent with the pathophysiology of uncontrolled asthma, in which persistent airway inflammation and nasal congestion promote mouth breathing. In our sample, children with uncontrolled asthma were more likely to exhibit mouth breathing, possibly because chronic nasal obstruction limits nasal airflow. These results align with the findings of Santos et al. [43], and a higher prevalence of mouth breathing among asthmatic children has been reported in the literature [43–46].

Mouth breathing has a recognized impact on occlusion, disrupting orofacial muscle balance and influencing dental and skeletal development, potentially resulting in malocclusions such as increased overjet, open bite, and crossbite [47].

Parental education and dental attendance were considered as potential confounding factors. In this study, fathers’ education levels differed between the two groups, while parental occupation showed no notable variation, likely due to the similar sociodemographic background of participants. Healthy children demonstrated more regular dental attendance than asthmatic children, who tended to visit only when experiencing pain or treatment needs. This lower attendance among asthmatic children may be explained by the increased burden of medical appointments related to asthma management, which can limit families’ ability to prioritize routine dental care [36, 38–40]. Although fathers’ education level differed between groups, it was not included in the final multivariable model. Given the modest sample size and limited number of outcome events, the inclusion of additional covariates could have resulted in model overfitting and unstable estimates. Therefore, clinically essential variables related to asthma status were prioritized in the adjusted analysis. Nevertheless, residual confounding cannot be entirely excluded, and this should be considered when interpreting the findings.

Regarding dental attendance specifically, only 22.7% of asthmatic children had visited a dental care center in the past year. Among these, the primary reason for attendance was treatment or pain relief, including conditions such as pulpitis, abscess, and cellulitis. This pattern indicates that dental attendance in this group is predominantly problem-driven rather than preventive. The low rate of regular attendance may be attributed to prioritization of asthma management, particularly in uncontrolled cases, as well as limited caregiver awareness of the potential oral health implications of asthma, including medication effects and mouth breathing. This significant difference in dental attendance between the study groups may have acted as a confounding factor influencing both oral health status and occlusal development.

Additionally, the inclusion of siblings in the control group may have introduced shared environmental and genetic influences, which may have attenuated the observed differences between groups, resulting in more conservative estimates.

In the present study, asthmatic children presented a significantly greater prevalence of increased overjet and Class II malocclusion (right canine relationship). The observed asymmetry in occlusal relationships between the right and left sides is likely attributable to developmental variations inherent to the mixed dentition stage, during which eruption and exfoliation may occur asynchronously between sides. Such physiological variability can result in transient occlusal discrepancies. Moreover, disturbances in arch integrity associated with local factors, such as interproximal caries or premature loss of primary teeth, may lead to space reduction and mesial drift of adjacent teeth, thereby contributing to occlusal imbalance.

Some occlusal parameters did not differ significantly between groups despite variations in mouth breathing. This may be due to multifactorial influences on occlusion, including genetic and developmental factors, as well as compensatory adaptations in craniofacial growth that may mask the effect of mouth breathing. Additionally, the sample size may limit the detection of subtle differences in certain traits.

Regarding transverse occlusal findings, no cases of posterior crossbite were observed in either group (0%). This may be related to sampling variation within this study population.

These findings agree with those of Zetina et al. [48], who reported a predominance of Class I molar relationships, followed by Class II and Class III relationships. Conversely, Kumar and Nandlal [49] reported a greater prevalence of posterior crossbites, whereas Tanaka et al. [50] reported that early-onset asthma (before 12 months of age) was associated with increased overjet and crossbite. These findings are relevant to our study, which included the assessment of craniofacial characteristics in asthmatic children.

The available evidence on salivary profiles in this population remains limited and inconclusive. In the present study, unstimulated whole saliva was used, given its clinical relevance and ability to reflect resting glandular function [36]. No significant differences were found between the groups in terms of salivary flow rate, pH, buffering capacity, or total antioxidant capacity. Furthermore, no correlations were detected between these salivary parameters and the use of inhaled corticosteroids or Montelukast.

Interestingly, the buffering capacity measured at pH 6.00 and 5.00 (NaHCl titration) was significantly greater in asthmatic children, despite similar total NaHCl volumes between the groups. One possible explanation is that asthmatic children, particularly those with chronic exposure to inhaled medications, may exhibit subtle alterations in salivary composition, although these changes are not consistently reflected across all salivary parameters. These findings may reflect potential variations in salivary buffering systems, although the exact underlying mechanisms remain unclear [51].

Additionally, variations in inhaled corticosteroid doses, dietary habits, and environmental factors could further contribute to the absence of significant differences in salivary parameters between groups.

The total antioxidant capacity (TAC) of saliva reflects the ability of salivary antioxidants to neutralize reactive oxygen species (ROS), which are known to contribute to oral tissue damage and compromise dental biofilm formation, a key defense mechanism against oral disease [24–28]. In the present study, TAC levels were slightly greater in healthy children (median 0.90 mM/L, IQR 0.98) than in asthmatic children (median 0.68 mM/L, IQR 0.76), although this difference was not statistically significant (*P* = 0.11). This finding suggests that, despite the potential oxidative stress associated with chronic asthma and long-term inhaled medication use, the overall salivary antioxidant capacity may remain relatively preserved in school-aged children.

Our findings align with those of Lumikarim et al. [52], who reported no significant difference in salivary pH between asthmatic and healthy children. Conversely, Arafa et al. [4] reported a reduction in salivary pH among asthmatic children. Similarly, Ersin et al. [53], Botelho et al. [54], and Fathima et al. [20] reported a decreased salivary flow rate and buffering capacity in asthmatic populations. Further studies evaluating specific salivary components are warranted to clarify these observations.

Overall, the findings of this study contribute to a better understanding of the oral health challenges faced by asthmatic children and underscore the importance of integrated dental and medical care in this population.

### Limitations

This study has several limitations. Its cross-sectional design precludes establishing temporal sequence or causality between asthma, mouth breathing, and malocclusion. The relatively small sample size limits generalizability. While mouth breathing was assessed using a mirror test and a questionnaire, a full otolaryngological evaluation was not performed. Residual confounding may remain despite matching for age and sex, due to differences in parental education, diet, oral hygiene, parafunctional habits, and environmental exposures. Larger longitudinal studies are warranted to confirm these associations and explore causal pathways.

## Conclusion

From this study, we can conclude that asthma is associated with mouth breathing, which may contribute to dental malocclusion. However, asthma and its medications showed no significant effects on the studied salivary parameters. Given the significant associations between asthma, mouth breathing, and occlusal traits observed in this study, it is evident that children with asthma are at increased risk of developing certain malocclusal traits. These findings highlight the need for early oral health interventions, respiratory monitoring, interdisciplinary care involving dentists, pediatricians, and allergists, and parental education to promote oral health and early detection of occlusal disorders.

## Supplementary Information

Supplementary Material 1.

Supplementary Material 2.

## Data Availability

All the data included in this study are available from the corresponding author upon request.

## Abbreviations

OS: Oxidative stress
TAC: Total antioxidant capacity
PPI: Proton pump inhibitors
ICS: Inhaled corticosteroids
SABA: Short-acting beta agonist
LABA: Long-acting beta agonist
MART: Maintenance and reliever therapy
AIR: Anti-inflammatory reliever) therapy
GINA: Global Initiative for Asthma
STROBE: Reporting of Observational Studies in Epidemiology
