# Supplementary material for: Impact of asthma on mouth breathing, occlusion and salivary parameters in a group of school-aged children: a cross-sectional study
Source: BMC Oral Health. 2026 May 18;26:907. doi: 10.1186/s12903-026-08537-7 (PMC13217853; doi:10.1186/s12903-026-08537-7)
Supplement: Supplementary file 1 — Supplementary Material 1. [file 12903_2026_8537_MOESM1_ESM.docx]

## (Supplementary File 1)

**Questionnaire for children with Asthma**

| **1** | **Sociodemographic Data** | |
| --- | --- | --- |
| **A.** | **Demographic data:** | |
| - Name: | | |
| - Age   - 6 y - 7 y - 8 Y - 9Y | | - 10 Y - 11 Y - 12 Y |
| -Gender:   - Female - male | | |
|  |  |  |
| **B.** | **Parental information :** | |
| - Parental occupation:   - Manual workers   (farmer, worker, private, self-employed ,unemployed)   - Non-manual workers   ( professor , doctor, government officer, teacher and manager). | | |
|  |  |  |
| - Parental education:   - non educated - Primary - Secondary - University (undergraduate / postgraduate) | | |
|  |  |  |
|  |  |  |

| **2** | **Dental history (** |
| --- | --- |
| **A.** | **Previous dental visits :** |
| - During past 12 month:   - Yes - No | |
| - Reason for that visit:   - Follow up and checkup - Treatment and pain relief | |

| **3.** | **Medical history** |
| --- | --- |
| **A.** | **Asthma type: in the past week has the patient had:** |
| i. Daytime asthma symptoms more than twice/week:   - Yes - No | |
|  |  |
| ii. Any night waking due to asthma:   - Yes - No | |
| iii. SABA* reliever for symptoms more than twice/week:   - Yes - No | |
| iv. Any activity limitation due to asthma:   - Yes - No | |
| - According to the last 4 questions Asthma is: (figure 4)   - well controlled (none of the last 4 Question) - partially controlled (1-2 the last 4 Question) - Uncontrolled (3-4of the last 4 Question ) | |

| **B.** | **The patient’s current treatment :** |
| --- | --- |
| **i.** | **Medication** |
| **1.** | **Inhaled Corticosteroids (ICS):** |
| - Current type, dose and frequency:  (e.g. fluticasone 125 2x2: dose is 125 mcg taken 2 puffs every 12h) | |
|  |  |
| - Duration of treatment: overall days since the start of therapy: | |
|  |  |
| - Cumulative doses since start of therapy : | |
|  |  |
| **2.** | **Reliever:** |
| - Type:  - SABA*: short acting beta agonist   - LABA*: long acting beta agonist     - SABA* - LABA* | |
|  |  |
| - Frequency of use: | |
| - Number of canisters per month : | |
| - Reliever use as:  - MART*: maintenance and reliever therapy) - AIR*: anti-inflammatory reliever) therapy   - Reliever only - a part of MART* - AIR* | |

| **3.** | **Systemic steroids :** |
| --- | --- |
| - How frequent in the last year: | |
| - Doses and duration: | |
| - Cumulative days : | |
|  |  |
| **4.** | **Montelukast:** |
| - Dose: | |
|  |  |
| - Form:   - Tablets - Chewable tablets - Sachets | |
|  |  |
| - Duration of use: | |
| - Cumulative days  : | |
| **ii.** | **Inhalation** |
| - Inhalation technique:   - Correct - Incorrect | |
|  |  |
|  |  |

| **C.** | **Symptoms of associated morbidities or exposures:** | |
| --- | --- | --- |
| i. Exposure to smoking:   - Yes - No | |  |
| ii. Exposure to industrial materials\noxious chemicals, dust, house animals and pets:   - Yes - No | | |
| iii. Symptoms of allergic rhinitis:   - Yes - No | | |
| iv. Eczema:   - Yes - No | | |
| v. Food allergy:   - Yes - No | | |
| vi. Receiving proton pump inhibitors (PPI):   - Yes - No | | |
| vii. Open mouth during sleep:   - Yes - No | | |
| ix. If yes to above question Open mouth during sleep:   - Transient with colds - In all sleeps - none | | |
|  |  |  |
| x. Snoring during sleep:   - Yes - No | | |
| xi. If yes to above question Snoring during sleep:   - Transient with colds - In all sleeps - none | | |

**Questionnaire for healthy children**

| **1** | **Sociodemographic Data** | |
| --- | --- | --- |
| **A.** | **Demographic data:** | |
| - Name: | | |
| - Age   - 6 y - 7 y - 8 Y - 9Y | | - 10 Y - 11 Y - 12 Y |
| -Gender:   - Female - male | | |
|  |  |  |
| **B.** | **Parental information :** | |
| - Parental occupation:   - Manual workers   (farmer, worker, private, self-employed ,unemployed)   - Non-manual workers   ( professor , doctor, government officer, teacher and manager). | | |
|  |  |  |
| - Parental education:   - non educated - Primary - Secondary - University (undergraduate / postgraduate) | | |
|  |  |  |
|  |  |  |

| **2** | **Dental history** |
| --- | --- |
| **A.** | **Previous dental visits :** |
| - During past 12 month:   - Yes - No | |
| - Reason for that visit:   - Follow up and checkup - Treatment and pain relief | |
