# Supplementary material for: Impact of asthma on mouth breathing, occlusion and salivary parameters in a group of school-aged children: a cross-sectional study
Source: BMC Oral Health. 2026 May 18;26:907. doi: 10.1186/s12903-026-08537-7 (PMC13217853; doi:10.1186/s12903-026-08537-7)
Supplement: Supplementary file 2 — Supplementary Material 2. [file 12903_2026_8537_MOESM2_ESM.docx]

Supplementary file

Title **Impact of asthma on mouth breathing, occlusion and salivary parameters in a group of school-aged children: A cross-sectional study**

Statements :

This study does not include any Gel or Blots images .
